# Radiomics as an emerging tool in the management of brain metastases

**DOI:** 10.1093/noajnl/vdac141

**Published:** 2022-09-06

**Authors:** Alexander Nowakowski, Zubin Lahijanian, Valerie Panet-Raymond, Peter M Siegel, Kevin Petrecca, Farhad Maleki, Matthew Dankner

**Affiliations:** Rosalind and Morris Goodman Cancer Institute, McGill University, Montreal, Québec, Canada; McGill University Health Centre, Department of Diagnostic Radiology, McGill University, Montreal, Québec, Canada; McGill University Health Centre, Department of Diagnostic Radiology, McGill University, Montreal, Québec, Canada; Rosalind and Morris Goodman Cancer Institute, McGill University, Montreal, Québec, Canada; Montreal Neurological Institute-Hospital, McGill University, Montreal, Québec, Canada; Department of Computer Science, University of Calgary, Calgary, Alberta, Canada; Rosalind and Morris Goodman Cancer Institute, McGill University, Montreal, Québec, Canada

**Keywords:** radiomics, brain metastases, radiology, artificial intelligence

## Abstract

Brain metastases (BM) are associated with significant morbidity and mortality in patients with advanced cancer. Despite significant advances in surgical, radiation, and systemic therapy in recent years, the median overall survival of patients with BM is less than 1 year. The acquisition of medical images, such as computed tomography (CT) and magnetic resonance imaging (MRI), is critical for the diagnosis and stratification of patients to appropriate treatments. Radiomic analyses have the potential to improve the standard of care for patients with BM by applying artificial intelligence (AI) with already acquired medical images to predict clinical outcomes and direct the personalized care of BM patients. Herein, we outline the existing literature applying radiomics for the clinical management of BM. This includes predicting patient response to radiotherapy and identifying radiation necrosis, performing virtual biopsies to predict tumor mutation status, and determining the cancer of origin in brain tumors identified via imaging. With further development, radiomics has the potential to aid in BM patient stratification while circumventing the need for invasive tissue sampling, particularly for patients not eligible for surgical resection.

## Brain Metastases: Current Approaches

Brain metastases are the most common adult brain tumor in adults, occurring in 20–40% of patients with metastatic cancer.^1,2^ From the time of diagnosis, patients with BM experience a median survival of less than 12 months.^1,2^ BM most commonly originate from primary tumors of the lung, breast, skin, kidney, and gastrointestinal system.^1,3^ Prognosis and therapeutic approaches are guided by several prognostic features, including performance status, the presence of extracranial metastases, type of primary cancer, tumor-mutational status in the form of molecular markers, and number and extent of BM.^1,4,5^ Notably, the mutation status of a tumor will often predict patient outcome and aid in treatment stratification.^1^ The invasion pattern of surgically resected BM and the presence of leptomeningeal lesions can also serve as relevant prognostic features.^6,7^

Current modalities for treating BM include surgical resection, stereotactic radiosurgery (SRS), whole-brain radiotherapy (WBRT), and systemic therapies, including chemotherapy, targeted therapies, and immunotherapies.^4,8-10^ Surgical resection is the first-line management of large and symptomatic BM.^11–13^ However, surgery is often not possible for patients with extensive extracranial disease burden, multiple anatomically distant brain metastases, metastases in eloquent brain areas, and leptomeningeal involvement.^14^

There is a growing impetus to stratify patients with BM to novel and personalized therapies, given the high rates of postoperative recurrence,^12,13,15–17^ radioresistance,^16^ and chemo-resistance^18^ associated with poor overall survival. Magnetic resonance imaging (MRI) and computed tomography (CT) are the modalities frequently used to visualize and diagnose tumors within the central nervous system.^19^

Medical imaging provides valuable information for clinicians to diagnose and subsequently manage BM. Expert analyses of images can allow for identifying lesions, tumor size, and number of metastases. Radiologists can frequently provide an informed opinion on whether a given lesion is metastatic or primary and can even predict the type of primary tumor.^20^ Improvements in medical image analysis technologies have begun to identify additional information within images, using novel imaging-based biomarkers that could be used to improve the accuracy of predicting clinical endpoints. It has become clear that medical images contain a plethora of quantitative, clinically relevant data that is often missed using traditional interpretation.^21–24^ This has led to the emergence of the field of radiomics that provides methodologies to extract additional quantitative information from images and identify features that may otherwise be too nuanced, small, or impractical to be detected by humans.^25^ The manner in which these quantitative analyses may help clinicians in the management of BM is thus an active area of research.

## Radiomics as a Clinical Tool

Radiomic analyses involve quantifying the relationships between voxels or pixels within an image^26–28^ assuming that small variations in pixel/voxel intensity, position, and density can serve as prognostic and predictive biomarkers for patient stratification.^29^

**Figure 1.**
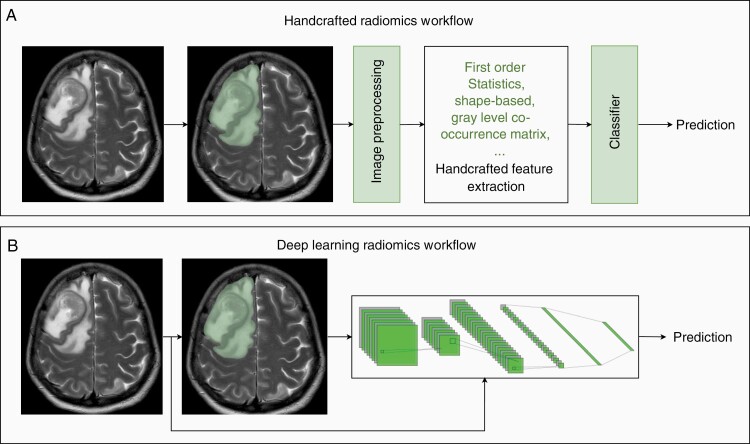
Radiomics pipelines utilizing a T2-weighted MRI image of a patient with BM, alongside the segmentation for the image. (A) A conventional handcrafted radiomic workflow in which images acquired from patients are manually, automatically, or semi-automatically segmented/contoured to delineate regions of interest. After applying perprocessing steps, such as normalization and denoising, the outlined tumors will undergo feature extraction using mathematical features such as first-order statistics, shape-based, and gray level co-occurrence matrix. After feature extraction, a classification model could be developed to predict the outcome of interest. (B) In a DL-based radiomic workflow, features are learned by a DL model using the available data for model training. Most often, a DL model consists of two components: a deep feature extractor followed by a classifier. The deep feature extractor component most often is a convolutional neural network (CNN), and the classifier component of the model is a shallow fully connected neural network. DL-based classification models often conduct a whole-image analysis and bypass the need for tumor segmentation. However, manually, automatically, or semi-automatically acquired segmentations could also be used in a DL-based workflow.

A radiomic classification pipeline can be outlined in 4 steps: (1) image acquisition, (2) image segmentation to isolate the region of interest, (3) image preprocessing, (4) feature extraction, and (5) prediction and classification of clinical outcomes by a classifier (Figure 1A and B).^30,31^

MRI is the standard imaging modality for diagnosis and monitoring of BM, with most of the existing BM radiomics literature utilizing MR images. In addition, CT and positron emission tomography (PET) images can also be used to image BM. For MRI, such analyses can be accomplished on standard MRI—using either T1- or T2-weighted (T1/2W) images—or using quantitative imaging techniques, such as fluid-attenuated inversion recovery (FLAIR), diffusion-weighted imaging (DWI), and perfusion-weighted imaging (PWI), which allow for more data to be used by radiomic models for more accurate predictions.

Tumor segmentation can be achieved through manual contouring or automated segmentation to delineate areas of interest computationally. Despite its simplicity, manual contouring is tedious and time consuming. It also suffers from inter-observer and intra-observer variability.^32,33^ As such, automated approaches to tumor segmentation have been developed.^34,35^ The increased use of deep learning (DL) in radiomic pipelines means that many models will skip tumor segmentation entirely, opting for whole-image analysis instead.^21^ In fact, DL models can be integrated to complete tumor localization and image segmentation, feature extraction, and classification (Figure 1B).^36–38^ Deep convolutional neural network models have been widely used for tumor segmentation.^39^ U-Net architecture and its variants are among the most commonly used models for tumor segmentation.^40^ In fact, conventional machine learning (ML) and DL U-Net models proposed for glioma segmentation have been systematically reviewed.^39^ They reported that deep learning models such as U-Net have the potential for deployment in a clinical setting.

Image preprocessing is a critical step between segmentation and feature extraction. It serves to create consistency amongst images prior to radiomic analyses. There is limited research on which preprocessing steps result in the most reproducible radiomic analyses. However, studies commonly use preprocessing steps such as voxel resampling and denoising. Resampling makes voxel dimensions consistent across images from all patients in a dataset. Resampling increases the robustness of radiomic features and often aids in their generalizability across datasets.^41,42^ Denoising aims to remove noise, defined as randomly distributed intensities unrelated to biological features. Denoising could improve the robustness of radiomic features.^42^ In studies where clinical images are compared across timepoints, image registration is essential, such that anatomical structures are lined up, permitting comparison of extracted radiomic features.^43^ However, the type of registration algorithm used could affect the repeatability of radiomic features.^43^

Processed images are subjected to feature extraction, whereby the intrinsic relationships between pixels/voxels are quantified and used to derive associations with clinical outcomes. Traditionally, feature extraction relied on “handcrafted” analyses, where quantitative features are defined based on first-order statistics, shape, and texture.^21^ DL-based algorithms have been increasingly used for image analysis and feature extraction.^21,44^ During this process, several hundred to a few thousand features are extracted. Classifiers combine extracted features and occasionally other clinically relevant data (eg, age, sex, and performance status). As such, classifiers can rely on either statistical methods or artificial intelligence (AI)-based approaches to predict clinical outcomes.^21^ Notably, ML approaches are particularly well suited for creating such prediction models since they can learn over time to improve prediction accuracy and are more suited for handling high-dimensional features. The implementation of AI in radiomics has several distinct advantages for quantitative image analysis. The use of AI-based classification permits the analysis of numerous radiomic features on large-patient datasets. Thus, clinical decision-making is based on a radiomic-signature, rather than the isolated predictive power of a single radiomic feature. ML-based classifiers make clinical predictions based on training data sets. “Deep”-extracted features—ie features extracted by DL models—can often be much more nuanced than those found through handcrafted analysis.^21^

A particular challenge for classifiers in BM-specific radiomics is the inherent clustering of radiomics features due to their high correlations between these features. Notably, tumors may be segmented as sub-regions, or patients may present with several lesions, leading to multiple observations per patient. The clustering resulted from the high correlation of these features can bias estimates of classifier performance, although no standard solution to this has been presented to date.^45^ When developing machine learning models, it is essential to avoid distributing data points for a patient across training and test sets as such scenarios violate the independent assumption, which states that data used for model training and evaluation should be independent. It has been empirically shown that violating the independent assumption could lead to a substantial but superficial and misleading boost in model performance.^46^ When analyzing multiple regions of interest within the same patient, calculating patient-level performance measures could prevent the bias introduced by multiple predictions associated with these regions of interest for some patients.^47^ However, this approach is not always applicable since the multiple predictions in multi-region analysis, eg, analyzing several lesions within the same patient, do not always need to be consistent. For example, some lesions within a patient might be malignant and some benign. Consequently, patient-level prediction is not always medically informative or relevant. Notably, variance adjustment, logistic random-effects models, and generalized estimating equations can be applied as other statistical methods to adjust for the high correlation in clustered data when calculating sensitivity and specificity.^47^

**Figure 2.**
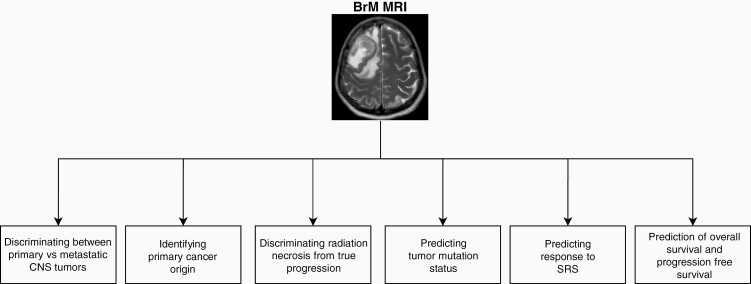
Current applications of radiomics in brain metastases (BM) management. Radiomics has emerged as a powerful tool in the personalized management of brain metastases (BM). This includes discriminating BM from primary central nervous system (CNS) tumors, identifying the site of primary cancer of origin, discriminating radiation necrosis from recurrence, predicting tumor mutation status, and predicting patient response to stereotactic radiosurgery (SRS).

Radiomic-identified biomarkers are not always fully explainable so that the quantitative values for each feature can be understood and interpreted by humans. This issue is more profound for DL models. However, the lack of interpretability of these models does not mean that they are not generalizable or reproducible. There have been efforts to standardize radiomic-derived biomarkers identified across different groups, creating defined biomarkers that could be generalized across software from different institutions.^41^ The Image Biomarker Standardization Initiative (IBSI) aimed at producing and validating reference values for radiomics features enabling the verification of radiomics software to increase the reproducibility of radiomics studies and facilitate model deployment in clinical settings.^41^ Despite these initiatives, variation in image acquisition, reconstruction, and segmentation might still lead to a lack of generalizability in radiomics studies. In multicentric or multiscanner settings, features extracted by standardized radiomics software might lack reproducibility due to sources of variations such as different scanner types, field strengths, or acquisition protocols. The role of biomarkers in radiomics is not limited to extracting features that may predict factors such as overall survival, as radiomic analysis may lead to the efficient and noninvasive identification of known biomarkers. For instance, prediction models can often accurately determine factors such as tumor mutation status and proliferative index (KI67 immunohistochemistry status).^48^ Radiomic and quantitative image analysis have become increasingly relevant tools in neuro-oncology. Imaging is continually a part of patient care, and radiomic analyses exploit this existing data to provide further noninvasive predictions pertaining to patient survival, mutation status, and other factors that can help stratify patients into a novel and more personalized treatment plans (Figure 2).

### Radiomics as a Tool to Predict Response to Stereotactic Radiosurgery

SRS is the standard of care for patients with 1–4 BM, less than 3 cm in diameter.^49–53^ For patients with more than 4, WBRT is the current standard of care with emerging data suggesting that up to 10 BM can be successfully treated with SRS.^54–56^ Unfortunately, not all patients are responsive to radiation therapy, with large BM rarely experiencing prolonged clinical benefit.^57–62^ Predicting response to SRS is challenging, and disease progression may only be apparent through imaging several months after treatment. Using serum-based biomarkers to predict sensitivity is promising but an approach in the early phases of development.^63^ Radiomic tools may be able to effectively predict patient response to radiotherapy using medical images alone.

Several studies have used radiomics models to predict patient response to SRS (summarized in Table 1).^64–67^ In one study, contrast-enhanced (CE) T1W and T2 FLAIR alongside a support vector machine was able to predict overall response, as well as response at 6- and 12-month time points based on early images of BM.^66^ The group retroactively separated patients and tumors into 2 cohorts: those predicted to show local control and those predicted to show local failure following SRS treatments. Tumors and patients predicted to experience local failure demonstrated significantly lower control rates and shortened overall survival. Other groups have focused on specific radiomic features as predictive biomarkers for response to SRS. Notably, both tumor enhancement volume and zone percentage were found to act as prognostic factors for BM local control in SRS-treated patients.^68,69^

**Table 1.** Radiomics as a Predictor of SRS Response

Study	Imaging modality	Study size	Classification type	External Testing/validation dataset	Non-Radiomic/clinical information used?	Model performance/results
Jiang et al.^71^	T1W, CE-T1W, T2W, T2-FLAIR.ADC, CBV, T1-MPRAGE	137 patients	Random forest	Yes	Gender, lung cancer subtype	AUC = 0.852
Gutsche et al.^67^	CE-T1W, T2W, FLAIR	150 patients	Random forest	Yes	Semantic features: contrast enhancement patterns classified as homogenous, heterogenous, or necrotic ring like	AUC = 0.74
Wang et al.^70^	CE-T1W	28 patients	Logistic regression	No	Radiation dose maps	AUC = 0.82 Standard deviation = 0.09
Huang et al.^69^	CE-T1W	161 patients	Consensus clustering to identify significant radiomic features	N/A	Age, Sex, EGFR mutation, KPS score, tumor location, tumor volume, prior chemotherapy, chemotherapy type	Zone percentage is associated with response to gamma-knife radiosurgery: MVCPHM- HR = 0.712, *P* = .022. MVCSPHM—HR = 0.699, *P* = .014
Kawahara et al.^72^	CE-T1W	54 patients	Neural net	Yes	No	AUC = 0.87
Mouraviev et al.^64^	CE-T1W, T2-FLAIR	87 patients	Random forest	No	Radiation prescription dose, ratio of the prescription dose and maximum dose for the lesion, maximum axial tumor diameter on CE-T1W, number of metastases, primary tumor site, and previous WBRT	AUC = 0.793 95% CI = 0.792–0.795
Della Seta et al.^68^	CE-T1W	48 patients	N/A	N/A	No	Univariable analysis of enhanced tumor volume as predictor of SRS response: *P* = .005 Hazard Ratio = 0.372 95% CI = 0.186–0.744
Karami et al.^66^	CE-T1W, T2-FLAIR	100 patients	Support vector machine	No	No	AUC (as estimated by 0.632 + rule) = 0.82
Cha et al.^73^	CT	89 patients	CNN	Yes	No	AUC = 0.856 95% CI = 0.702–1

ADC: apparent diffusion coefficient, AUC: area under the curve, CBV: cerebral blood volume, CE: contrast-enhanced, CI: confidence interval, CT: computed tomography, FLAIR: fluid-attenuated inversion recovery, MPRAGE: magnetization-prepared 180 ° C radio-frequency pulses and rapid gradient-echo, T1/2W: T1/2 weighted, HR: hazard ratio, MVCPHM: multivariate cox proportional hazards model, MVCSPHM: multivariate cause-specific proportional hazards model.

The information selected here illustrates the best-performing model. Model performance portrayed on testing data sets, if available.

One study predicting SRS-responsiveness incorporated a large number of clinical features, such as radiation dose, ratio of prescription dose to maximum dose for the lesion, tumor diameter, primary tumor site, number of metastases, and previous WBRT treatment with CE-T1W and T2-FLAIR images alongside a random forest-based classifier.^64^ Although clinical features alone performed poorly with an area under curve (AUC) value of 0.669, performance was enhanced when combining radiomic features with clinical information generating an AUC of 0.793. Similarly, another study incorporated information relating to radiation dose in BM patients treated with SRS in CE-T1W images.^70^ The best-performing model using logistic regression analysis incorporated features relating to dose skewness, dose entropy, and dose minimum alongside other handcrafted radiomic features.

One group compared radiomic features to CE patterns defined by three radiologists as either homogenous, heterogeneous, or necrotic ring-like from CE-T1W, T2W, and FLAIR images as predictors of patient response to SRS.^67^ Their best-performing model integrated both radiomic and semantic features for classification by a random forest algorithm.

Multiparametric MRI also has the potential to predict patient response to SRS treatment. Numerous MRI modalities (Table 1), coupled with features extracted from both the tumor core and peritumoral edema, were used to generate a radiomic model.^71^ A random forest algorithm incorporated features from several different imaging modalities, including apparent diffusion coefficient (ADC) and cerebral blood volume (CBV) maps, were significant for predicting patient response.^71^

Notably, 2 studies have included DL in their radiomic pipeline for the prediction of response to SRS.^72,73^ One group classified patients based on predicted response to gamma-knife radiosurgery using a 10-layered neural network from CE-T1W images.^72^ The model showed an accuracy of 78%; however, with a testing set of only 9 patients, the generalizability to larger data sets may be challenging. Conversely, the second group applied a DL for both feature extraction and classification on CT images of BM.^73^ They created “ensemble” models that consisted of 10 differently trained and validated convolutional neural networks (CNNs), resulting in their highest predictive power. Notably, CT images are used less frequently for the imaging of BM compared to MRI. Patients enrolled in the study were limited to a short 3-month follow-up. Thus treatment response did not account for pseudoprogression.

The body of literature describing radiomics-based predictions of SRS response is still developing. Future studies may focus on incorporating larger and multi-institutional data sets as models begin to become more generalizable for clinical use. Previous research in the field has shown that semantic features from contrast-enhanced MRI can act as prognostic features for SRS responsiveness.^74^ However, it is evident that there is a need for more biomarkers aiding in the identification of these non-responsive patients. As the radiomics field develops within this context, it will likely prove to be a useful approach for more personalized management of BM.

With the increasing prevalence of SRS use, and particularly with the addition of concomitant systemic therapies and longer patient survival, radiation necrosis has become an increasingly prevalent issue.^75^ Radiation necrosis is the death of parenchymal brain tissue following radiotherapy, with larger BM treated with a greater applied radiation dose being at higher risk.^75^ It is distinct from tumor recurrence or progression, in which radiation necrosis is not a malignant progress, despite exhibiting a high degree of overlap in symptoms and radiological appearance.^75^ Establishing strategies to accurately differentiate radiation necrosis from recurrence and tumor progression has the potential to significantly improve the care and quality of life of patients treated aggressively for lesions of equivocal significance.^75^ Notably, this could enable clinicians to halt further radiotherapy and potentially alleviate symptoms using drugs such as bevacizumab or corticosteroids.^76–78^

Several publications have focused on discriminating cases of radiation necrosis from local recurrence (LR) or tumor progression in BM patients (summarized in Table 2).^65,79–85^ This task remains very challenging for clinicians using traditional interpretations, and radiomic models will frequently outperform radiologists.^65,84^ Radiomics analyses can often discriminate radiation necrosis from true disease progression with AUC values generally above 0.80 (Table 2).

**Table 2.** Predicting Radiation Necrosis from Progression and Recurrence

Study	Imaging modality	Study size	Classification type	External testing/validation dataset	Non-radiomic/clinical information used?	Model performance
Chen et al.^82^	CE-T1W, T2-FLAIR	109 patients	Random forest	Yes	No	AUC = 0.71 95% CI = 0.51-0.91
Dohm et al.^80^	CE-T1W, FLAIR	73 patients	Multivariate logistic analysis	Yes	No	Mechanistic/biophysical modeling: AUC = 0.95 95% CI = 0.94-0.97 Radiomic features: AUC = 0.77 95% CI = 0.75-0.80
Cai et al.^85^*	FLAIR	149 patients	Multivariable logistic analysis	Yes	Interval between radiotherapy and diagnosis of brain necrosis, interval between diagnosis of brain necrosis and treatment with bevacizumab	AUC = 0.827 95% CI = 0.691-0.962
Hettal et al.^84^*	CE-T1W	20 patients	Bagging algorithm	No	No	AUC = 0.83 95% CI = 0.65-1
Hotta et al.^79^	MET-PET	41 patients	Radom forest	No	No	AUC = 0.98
Lohmannet al.^81^	FET-PET, CE-T1W	52 patients	Multivariate logistic regression	No	No	AUC = 0.86
Peng et al.^65^	CE-T1W, T2-FLAIR	66 patients	Support vector machine	No	No	AUC = 0.81
Zhang et al.^83^	T1W, CE-T1W, T2W, FLAIR	84 patients	Ensemble classifier: RUSBoost	No	No	AUC = 0.73

AUC: area under the curve, CE: contrast-enhanced, CI: confidence interval, CT: computed tomography, FLAIR: fluid-attenuated inversion recovery, FET: O-(2-[^18^F]-fluoroethyl)-l-tyrosine, MET: 11C-methionine, PET: positron emission tomography, T1/2W: T1/2 weighted.

The information selected here illustrates the best-performing models. Model performance portrayed on testing data sets, if available.

*The studies by Cai et al. and Hotta et al. predicted response in CNS-tumor patients with necrosis, not specific to BMs.

A recent study employed an integrated multiparametric radiomics, extracting features from combined CE-T1W and T2-FLAIR images to be classified by a random forest algorithm.^82^ Incorporating patients from two separate institutions and using a dedicated testing set aided their model’s potential generalizability. As such, these multiparametric approaches may show merit in future studies, aiding in clinical predictions.

The use of PET—particularly dynamic amino acid PET—has the potential to improve the ability to discriminate radiation necrosis from progression.^86–89^ For instance, a multivariate logistic regression-based classifier could accurately discriminate between radiation injury and true progression using combined O-(2-[^18^F]-fluoroethyl)-l-tyrosine (FET)-PET and MRI.^81^ Interestingly, prediction using strictly FET-PET features performed better than one using MRI alone. However, combining information from the 2 modalities resulted in their best-performing model with an accuracy of 89%. Additionally, 11C-methionine (MET)-PET scans have also been used to discriminate true progression from radiation necrosis.^79^ They did not limit their study to BM and included patients with gliomas. While the results using PET from both groups are promising, it is notable that FET and MET are not radiotracers currently used clinically for PET scans in lieu of ^[18F]^-Fluoro-2-deoxy-d-glucose (FDG).^90^

In the context of treating radiation necrosis, some patients show no benefit with bevacizumab treatment and occasionally even display worsening.^91^ To address this issue, one study used a multivariable logistic analysis classifier from FLAIR image features that could predict patients with responsiveness to bevacizumab as a treatment for radiation necrosis.^85^ This work was not specific to only patients with BM but rather to patients with all primary and secondary central nervous system (CNS) tumors exhibiting radiation injury. Their prediction model considered abundant clinical information such as the interval between radiotherapy and diagnosis of radiation necrosis, the interval between radiation necrosis onset and bevacizumab treatment, and features extracted from FLAIR imaging. A comparison a radiomics-based multivariate logistic analysis classifier to biophysical modeling of tumor growth showed the latter to be favorable in predicting radiation injury from CE-T1W and FLAIR images.^80^ Biophysical modeling of lesion growth allows for patient-specific mathematical prediction of tumor growth based on clinical images.^92^ The inclusion of mechanistic features extracted through biophysical modeling could be incorporated alongside radiomic features to improve prediction accuracy.

Overall, the studies describing radiomic models that discriminate between radiation necrosis and true progression in patients with BM show the potential for future clinical applications.

### Radiomics to Identify Brain Metastasis Mutational Status

With advances in systemic therapies often designed to target cancer-specific driver mutations, identifying genetic alterations within a patient’s tumor is of utmost importance for personalized management of BM. However, a patient’s BM can diverge substantially from their primary tumor of origin with respect to mutation status and frequently acquire or lose targetable genetic lesions.^93^ Performing next-generation sequencing, immunohistochemistry, and PCR-based assays on biopsies and surgical specimens from primary tumors or BM are used as the gold standards to identify tumor mutation status. With further development, radiomics has the potential to circumvent the need for invasive brain tissue sampling by acting as a virtual biopsy, particularly for those patients not eligible for surgical resection.

Applying radiomics to identify underlying tumor mutation is an emerging application within this field. This phenomenon is particularly notable in the context of non-small cell lung carcinoma (NSCLC) BM where patients can exhibit several different genetic mutations or rearrangements that are treatable with targeted agents. Frequently mutated genes include EGFR, ALK, KRAS, BRAF, HER2, ROS1, and RET.^94^

Several studies have emerged using radiomics as a “virtual biopsy” for EGFR mutation status in lung cancer patients, showing moderate performance in the prediction of EGFR mutation status with AUC values ranging between 0.75 and 0.95^95–97^ (Table 3). However, small sample sizes may hurt the generalizability of certain models clinically.^95–97^ A study attempted to use a random forest-based classifier to predict mutation status in lung cancer patients with BM using contrast-enhanced T1-weighted and T2-FLAIR MRIs evaluating, EGFR, ALK, and KRAS mutation status.^97^ These studies show promise for radiomics as a tool for managing NSCLC patients exhibiting BM.

**Table 3.** Radiomics as a Predictor of BM Mutation Status

Study	Imaging modality	Study size	Cancer types and mutations	Classification type	External Testing/Validation Dataset	Non-Radiomic/Clinical information used?	Model Performance
Ahn et al.^95^	CE-T1W	61 patients	Lung cancer: EGFR mutation status	Random forest	No	No	Small BrMs: AUC = 0.890 Large BrMs: AUC = 0.782
Chen et al.^97^	CE-T1W, T2-FLAIR	110 patients	Lung cancer: EGFR, ALK, KRAS mutation status	Random forest	No	Additional sites of metastases, number of tumors, volume of the tumor core, edema/tumor volume ratio, gender, race and smoking history	EGFR status: AUC = 0.912 ALK status: AUC = 0.915 KRAS status: AUC = 0.985
Park et al.^96^	DTI, CE-T1W	51 patients	NSCLC: EGFR mutation status	Radom forest	Yes	No	AUC = 0.765 95% CI = 0.638 – 0.889
Meißner et al.^102^	CE-T1W, T2W	59 patients	Melanoma: BRAF mutation status	Support vector machine	Yes	Patient age, gender, previous systemic therapy, time from first diagnosis, number of BrMs, volume, tumor location, symptoms, and Karnofsky performance status	AUC = 0.92
Shofty et al.^101^	CE-T1W	54 patients	Melanoma: BRAF mutation status	Support vector machine	No	Age, gender, metastasis size	AUC = 0.78

AUC: area under the curve, CE: contrast-enhanced, CI: confidence interval, CT: computed tomography, DTI: diffusion tensor imaging, NSCLC: non-small cell lung cancer, SCLC: small cell lung cancer, T1/2W: T1/2 weighted.

The information selected here illustrates the best-performing models. Model performance portrayed on testing data sets, if available.

BRAF mutation status is an important biomarker for personalized management of metastatic melanoma.^98–100^ In one study, tumor location, shape, first-order, and second-order features from CE-T1W MRIs, alongside a support vector machine classifier were used to predict BRAF mutation status in melanoma-derived BM.^101^ Akin to previous studies predicting BM mutation status, this model showed an accuracy of 78%, although the study contained a limited number of samples. Another group has also published a support vector machine based classifier that could specifically predict the presence of BRAF V600E mutation in melanoma patients with BM with an accuracy of 86%.^102^ The classifier incorporated features from CE-T1W images, T2W images, and clinical information.

Radiomics as a tool for virtual biopsy of BM is still with very recent and emerging applications. Studies in the context of both lung and melanoma BM have shown that there is potential for future clinical use. In the context of glioblastoma and other primary brain tumors, there is a large body of work demonstrating the applicability of radiomics for determining alterations in MGMT promoter methylation^103–106^ and mutations in IDH,^107,108^ ATRX,^109^ TP53,^108^ and EGFR.^110^ Future models could thus target predicting multiple genetic alterations within a single lesion.

There is impetus for future studies to focus on incorporating larger and multi-institutional data sets to further illustrate the potential and generalizability of radiomics in this context. Furthermore, it remains to be seen whether radiogenomics approaches can predict individual mutations in single oncogenes with many different potential driver mutations beneficial to targeted therapies, such as BRAF.^111,112^

### Applying Radiomics to Differentiate Between Primary Brain Tumors and BM

With MRI, solitary BM in the absence of a known primary tumor can be difficult to differentiate from primary tumors of the CNS origin,^113^ even though the increased use of quantitative MRI sequences has improved the accuracy of these diagnoses.^114,115^ Management of primary brain tumors differs substantially from that of BM, and misdiagnoses of either glioblastoma as BM or vice-versa do occur and are of significant consequence for affected patients.^12,113^ In this regard, multiple studies have applied radiomics to develop tools capable of differentiating patients with BM from those with primary CNS tumors (Table 4).^116–122^ The utility of radiomics to discern primary from metastatic brain lesions was underscored by demonstrating that two such models outperform neuroradiologists.^119,123^

**Table 4.** Differentiation Between Primary CNS Tumors and BM

Study	Imaging modality	Study size	Classification type	External testing/validation dataset	Non-radiomic/clinical information used?	Model performance
Mărginean et al.^122^	CT	36 patients	Univariate and multivariate statistical analyses	N/A	No	AUC = 0.992 95% CI = 0.903-1
Bae et al.^123^	T2W, CE-T1W	166 patients in training cohort, 82 patients in validation cohort	Deep learning neural net	Yes	No	AUC = 0.956 95% CI = 0.918-0.990
Dong et al.^118^	T1W, T2W, CE-T1W	120 patients	Five classifiers reach agreement: decision tree, neural network, support vector machine, k-nearest neighbor, naïve bayes	Yes	No	Accuracy = 0.77 Specificity = 1.00
Ortiz-Ramon et al.^116^	CE-T1W	50 glioblastoma patients, 50 BrMs patients	Multilayer perceptron	No	No	AUC = 0.912 Standard deviation = 0.060
Artzi et al.^117^	CE-T1W	439 patients	Support vector machine	Yes	Patient age, gender, and weight	AUC = 0.96
Chen et al.^120^	CE-T1W, T2W, T2-FLAIR	134 patients	Distance correlation and logistic regression	Yes	No	AUC = 0.80
Qian et al.^119^	T1W, CE-T1W*, T2W	412 patients	Support vector machine	Yes	No	AUC = 0.90

AUC: area under the curve, CE: contrast-enhanced, T1/2W: T1/2 weighted, FLAIR: fluid-attenuated inversion recovery, CT: computed tomography, CI: confidence interval. The information selected here illustrates the best-performing models. Model performance portrayed on testing data sets, if available.

*Tumor segmentation was only performed on CE-T1W images in the study by Qian et al.

Current literature primarily uses handcrafted features alongside classical ML approaches.^116–122^ Despite this focus on more “traditional” radiomic approaches in the field, DL-based approaches show promise, as one group demonstrated increased predictive power in comparison to their ML-based classifier.^123^ Additionally, models discriminating between glioblastoma and BM is not limited to MRI, as other groups have used CT to similar effect.^122^

Several groups working on this application emphasized generalizability, through the use of *PyRadiomics that is* an open-source library allowing for standardized feature extraction, thus creating consistency across extracted features.^118,119,123,124^ Several groups also included external validation in their studies.^119,123^

Future radiomics models could thus serve as an important tool for clinicians attempting to distinguish between BM from primary CNS tumors. In the context of primary CNS tumors, radiomics has been applied to aid in grading gliomas^125,126^ and distinguishing glioblastoma from other CNS malignancies, such as primary central nervous system lymphoma (PCNSL).^127,128^ These studies could soon evolve to distinguish lower-grade gliomas and PCNSL from BM.

Primary cancer site of origin is unknown in up to 15% of patients with BM, and nearly 5% of BM patients still have an unknown primary tumor type after autopsy.^129^ This complicates management, as systemic therapies vary mainly depending upon the site of the primary cancer.^1^ Biopsies coupled with pathology work-up can provide critical information regarding the primary cancer origin of BM but are rarely used in this context, given their invasiveness.^130^ However, the application of imaging-based approaches for diagnosing primary tumor of origin would avoid invasive tissue sampling. Radiomics has begun to be applied in this setting, with the goal of rapid and noninvasive assessment of BM origin.

Several radiomics models have been developed with the goal of distinguishing primary origin sites based on images of existing BM (Table 5).^131,132^ One study aimed to discriminate between BM and glioblastoma primary lesions, alongside primary tumor origin; however, only focusing on tumors from breast and lung.^117^ The group extracted features from CE-T1W MRIs, classifying using a support vector machine algorithm. Another group used 3-dimensional features from T1W MRIs and a random forest algorithm for discriminating between BM from lung and breast cancer (accuracy of 86%) and BM from lung cancer and melanoma (accuracy of 86%).^132^ However, this model performed poorly in discriminating breast and melanoma-derived BM from one another with an accuracy of 56%. This study was limited to only these three primary cancers, which may limit generalizability, particularly in cases of patients with BM originating from colorectal, renal, or other primary sites of origin.

**Table 5.** Predicting Primary Cancer Origin Site

Study	Imaging modality	Study size	Cancer types	Classification type	External testing/Validation dataset	Non-Radiomic/Clinical information used?	Model performance
Zhang et al^133^	CE-CT	144 patients	NSCLC subtypes: adenocarcinoma, squamous cell carcinoma	Binary logistic regression	Yes	Age and sex	AUC = 0.828 95% CI = 0.738–0.918
Kniep et al^131^	T1W, CE-T1W, FLAIR	189 patients	SCLC, NSCLC, breast cancer, melanoma, gastrointestinal cancer,	Random forest	Yes	Age and sex	Discrimination of 3 cancer types: SCLC: AUC = 0.89 Breast cancer: AUC = 0.86 Melanoma: AUC = 0.83 Discrimination of 5 cancer types: SCLC: AUC = 0.76 Breast cancer: AUC = 0.78 Melanoma: AUC = 0.82 Gastrointestinal: AUC = 0.68 NSCLC: AUC = 0.64
Ortiz-Ramonet al^132^	T1W	38 patients	Lung cancer, breast cancer, melanoma	Random forest	No	No	Lung vs. breast: AUC = 0.963 SD = 0.054 Lung vs. melanoma: AUC = 0.936 SD = 0.070 Breast vs. melanoma: AUC = 0.607 SD = 0.180

AUC: area under the curve, CE: contrast-enhanced, CI: confidence interval, CT: computed tomography, FLAIR: fluid-attenuated inversion recovery, NSCLC: non-small cell lung cancer, SCLC: small cell lung cancer, T1/2W: T1/2 weighted.

The information selected here illustrates the best-performing models. Model performance portrayed on testing data sets, if available.

One study used T1W, CE-T1W, and FLAIR images alongside a random forest classifier to discriminate primary tumor of origin from BM images.^131^ The model accurately predicted BM originating from breast, small cell lung cancer (SCLC), and malignant melanoma. Prediction accuracy, however, was lower when for discerning patients with brain lesions originating from NSCLC and gastrointestinal cancers.

There have been efforts to use radiomics to distinguish subtypes within NSCLC BM. A binary logistic regression classifier could accurately distinguish NSCLC-originating BM as being either adenocarcinomas or squamous cell carcinomas using contrast-enhanced CT images.^133^

Future studies in this area would benefit from incorporating data sets for patients with “other” primary cancer types. While breast, melanoma, and lung cancers represent the most BM cases, the ability of models to recognize BM from gastrointestinal and kidney cancers, for instance, is essential for clinical applications in a primary tumor-agnostic patient population, as is seen in the real world.^1^ Despite this, the existing literature demonstrates that radiomics has the potential to identify primary cancer origin in BM.

### The Future of Radiomics for BM Management

Despite the progress made implicating radiomic models for the management of BM, there remains ample space for future development. A large proportion of radiomics research using MRI and CNS tumors has focused on glioblastoma, which thus hints at potential applications to BM. Such possibilities include the use of radiomic models to predict overall survival, progression-free survival, and local recurrence-free survival for patients with BM. Notably, both overall and progression-free survival have been the target of radiomics models for glioblastoma patients.^134^ Recently, several studies have emerged predicting overall survival in patients with BM, although these have remained specific to NSCLC and melanoma primary cancer types.^135–137^

Advances in the past years have shown that radiomics is capable of predicting mutation status subtyping, particularly in the context of NSCLC BM. Future research could similarly focus on breast cancer BM subtyping, which can be an important factor for patient outcomes upon diagnosis of metastatic spread.^138^ Additionally, factors such as KI67 expression, HIF-1α status, and the presence of microvasculature are prognostic factors for BM.^139,140^ In the context of glioblastoma, it has been shown that radiomics can predict KI67 status suggesting that this is a possibility for BM as well.^141,142^

Tumor invasiveness has been shown to be an important prognostic factor for overall survival and local-recurrence free survival in the context of BM.^7^ Developing a radiomics-based approach that could predict invasion patterns of brain metastases may be advantageous for clinicians with the further development of this biomarker, as has been shown for histopathological growth patterns of colorectal cancer liver metastases.^143^ Radiomic prediction of brain invasiveness has similarly been pre-operatively demonstrated in meningioma, suggesting promise for future applications in BM.^144,145^

Another development pertinent to BM-specific radiomic models is the inclusion of peri-tumoral imaging. Notably, in the context of meningioma, peritumoral edema volume has shown to be an important biomarker for predicting invasiveness.^145^ In the context of BM management, peritumoral images have seldom been used for analysis. Notably, peritumoral regions, including both edema and tumor lesion borders, were shown to contain important information for a model predicting BM response to SRS.^66^ Additionally, a study in patients with single BM showed that textural analysis of apparent diffusion coefficient (ADC) images derived from DWI could improve clinical risk models.^146^

Radiomics has proven to be a promising approach for the personalized management and stratification of patients with BM. However, as this tool moves toward the clinic, there are several important considerations and limitations to consider. It is evident that radiomic models will serve as an additional tool for clinicians rather than a standalone modality for diagnostics and prognostication. Moreover, despite the inherent advantages of high-order image analysis, it is not a complete replacement for traditional semantic analyses performed by radiologists, as evident by several publications outlined in this review.^64,67^ Multi-dimensional data integration will be a key feature in future radiomics studies. This will expand past clinically relevant data such as patient age, sex, and primary site of origin to also include pathologic and genetic information to improve model performance. Notably, any radiomic model used in practice needs to perform consistently. Reproducibility and generalizability remain an issue within the field as current studies often focus on small, single-institution datasets. Future studies should aim to incorporate larger and cross-institutional data sets to demonstrate the large-scale merit of radiomic models in BM management. Additionally, these studies may cultivate higher-performing models by using heterogeneous data sets that contain differences in patients and image acquisition. Notably, there have been efforts to standardize image acquisition across all radiomic studies in clinical trials to increase reliability with regard to tumor detection and measurement.^147^ These efforts have resulted in consensus recommendations for a standardized brain tumor imaging protocol that includes “minimum standard” and “ideal” imaging protocols which include the implementation of black-blood imaging.^147^ Furthermore, there should be an emphasis on using standardized, open-source image analysis packages, such as *PyRadiomics*, to ensure reproducibility.^41^

In summary, we outlined the emerging use of radiomics for the personalized management of BM. Radiomics has yet to reach the forefront of clinical management for patients with BM. However, as this field continues to evolve, particularly with large multi-institutional studies, such a possibility will become closer to reality.
